# Commentary: The second cut is the deepest

**DOI:** 10.1016/j.xjtc.2021.05.008

**Published:** 2021-05-14

**Authors:** Stephanie N. Nguyen, David Blitzer, Shepard D. Weiner, Hiroo Takayama

**Affiliations:** a Division of Cardiac, Thoracic and Vascular Surgery, Department of Surgery, Columbia University Medical Center/New York-Presbyterian, New York, NY; b Division of Cardiology, Department of Medicine, Hypertrophic Cardiomyopathy Center, Columbia University Medical Center/New York-Presbyterian, New York, NY

**Central Message**

Aggressive myectomy through transapical approach not only relieves basal and midventricular obstructions but also may augment diastolic dysfunction in hypertrophic cardiomyopathy.

**Figure:**
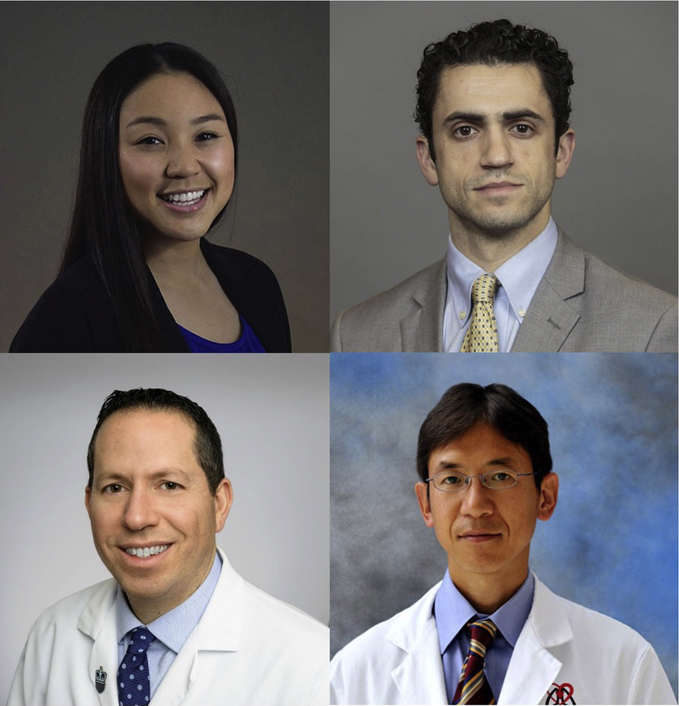
Stephanie N. Nguyen, MD, David Blitzer, MD, Shepard D. Weiner, MD, and Hiroo Takayama, MD, PhD

See Article page 71.

The video clip by Sun and colleagues^1^ demonstrates an impressive degree of cavity obliteration with an extremely thick interventricular septum measuring 48 mm. The patient had previously undergone transaortic septal myectomy to relieve left ventricular outflow tract (LVOT) obstruction at another institution with persistence of heart failure symptoms. A reoperative transapical myectomy was performed with significant improvement in left ventricular chamber size and diastolic function. This case poses at least 2 important questions.

First, how do we determine whether symptoms are secondary to LVOT obstruction or diastolic dysfunction? In reality, many patients suffer from a variable combination of both pathologies, and failure to recognize the latter may result in residual symptoms, even though transaortic septal myectomy is an excellent septal reduction therapy for relief of LVOT obstruction^2^^,^^3^ and can be performed with less than 1% mortality in experienced hypertrophic cardiomyopathy (HCM) centers.^4^ Of note, in a large, single-center series of 699 patients who underwent transaortic septal myectomy, 14.5% remained in class II and 4.5% in class III at mean follow-up of 6.2 ± 3 years.^5^ “Only” 81% improved to New York Heart Association class I. Clearly, LVOT obstruction may not be the only problem at hand.

Second, does an aggressive myectomy relieve diastolic dysfunction? There is a clear relationship between diastolic dysfunction and heart failure in nonobstructive HCM, and this should be evaluated with echocardiography and invasive hemodynamic catheterization to identify this larger group of patients with HCM. The Mayo group has championed a transapical approach. Their combined transaortic and transapical approach has been shown to abolish any cavitary obstruction and augment left ventricular diastolic function with good short-term outcomes.^6^^,^^7^ This is important, as untreated midventricular obstruction not only leads to significant functional impairment but is also associated with reduced survival and greater risk of ventricular arrhythmias.^8^ Fortunately, complex multilevel septal hypertrophy occurs only ∼5% of patients with HCM. However, their recent case series showed a larger purpose of transapical myectomy. In their series of 113 patients with apical HCM who underwent transapical myectomy, 76% of patients reported an improvement in symptoms with superior survival to patients with HCM listed for heart transplantation.^9^ These promising results suggest that an aggressive myectomy does relieve diastolic dysfunction. The presented case is an excellent illustration of this approach. Seeing is believing.

This 24-year-old male patient might have received a heart transplantation had he been treated elsewhere. While our own institutional data of 41 patients with end-stage HCM managed with heart transplantation suggest it is a good option,^10^ transplant comes with costs. It may be time for HCM and heart failure experts to listen to the message of the Mayo group, “long-term survival (after transapical myectomy) appears superior to those listed for heart transplantation.”^9^
